# Predicting adverse events for risk stratification of chemotherapy based stem cell mobilization in multiple myeloma

**DOI:** 10.1038/s41746-026-02394-y

**Published:** 2026-02-03

**Authors:** F. Schwarz, L. Levien, M. Maulhardt, G. Wulf, N. Brökers, E. Aydilek

**Affiliations:** 1 Department for Hematology and Medical Oncology, University Medical Center Göttingen, Göttingen, Germany; 2 Göttingen Campus Institute for Dynamics of Biological Networks (CIDBN), University of Göttingen, Göttingen, Germany; 3 Max Planck Institute for Dynamics and Self-Organization, Göttingen, Germany; 4 Department of Internal Medicine, Hematology, Oncology, Stem Cell Transplantation and Palliative Medicine, Protestant Hospital Bethel, University Hospital OWL, Campus Bielefeld-Bethel, Bielefeld, Germany

**Keywords:** Myeloma, Myeloma, Health care economics, Quality of life, Stem-cell therapies

## Abstract

Autologous stem-cell transplantation is a fundamental therapy for multiple myeloma. Although inpatient chemo-based stem-cell mobilization (SCM) is standard care in Germany, outpatient approaches could ease healthcare constraints. We analyzed 109 myeloma patients undergoing SCM and collection at the University Medical Center Göttingen for safety. We then trained machine learning models to predict adverse events (AEs) requiring hospitalization and to forecast AE onset timing for optimized ward management. In our cohort, 97% achieved successful collection, but 69% experienced severe AEs necessitating hospitalization. Simulations suggest a risk-stratified outpatient protocol could cut bed usage by at least one third without compromising safety. Classification models accurately predicted some AE types (e.g., elevated creatinine, ROC-AUC 1.0), though neutropenic fever remained challenging (ROC-AUC 0.67). Regression models forecast AE onset with a mean error of just over one day. These results outline a data-driven roadmap for safely adopting outpatient SCM and optimizing resource allocation in clinical practice.

## Introduction

Multiple myeloma (MM) is a rare malignant plasma cell disorder characterized by an abnormal clonal antibody production within the bone marrow^1^. Although advances in therapy have transformed MM into a manageable chronic condition, it is deemed an incurable condition for most patients^2^. The global incidence of MM is rising^3^, a trend attributed to demographic shifts, particularly due to an aging population^4^. This trend is therefore projected to add additional burden to our health care systems, necessitating the development of innovative treatment and management strategies to ensure sustainable, high-quality care for MM patients.

Autologous stem cell transplantation (ASCT) following induction therapy remains the standard of care in Germany for eligible MM patients^5^. It is a pivotal treatment choice for eligible patients in first remission, as it enhances bone marrow regeneration after high-dose chemotherapy and reduces relapse probability^6^. Even in older patients (≥70 years), ASCT shows a significantly higher progression-free survival compared to non-transplant strategies (41 vs. 33 months, respectively^7^). Stem cell mobilization (SCM) and collection (SCC) are critical steps preceding ASCT. Chemotherapy-assisted mobilization, particularly by cyclophosphamide in combination with granulocyte colony-stimulating factor (G-CSF), generally yields higher stem cell counts compared to G-CSF alone^8^. However, there is a lack of data for novel induction regimens, including anti-CD38 antibodies, or intermediate/high-dose cyclophosphamide regimens with or without etoposide.

A possible way to reduce stress on ward bed capacities would be outpatient regimens for SCM and/ or SCC. Nevertheless, inpatient mobilization is the standard of care in most centers in Germany. The feasibility of outpatient strategies depends on healthcare infrastructure, patient risk stratification and the ability to manage potential complications, particularly febrile neutropenia (FN)^9^. Thereby, a key challenge remains the early identification of patients requiring hospitalization due to severe adverse events (SAEs).

This study investigates the safety of SCM in MM patients with various types of mobilization protocols and drug doses in an inpatient setting. Based on our observations, we assess the feasibility of outpatient mobilization strategies by simulating and comparing different scenarios to develop concepts for potential future outpatient treatment. Furthermore, we evaluate machine-learning models to predict SAEs leading to hospitalization by introducing a multi-stage framework that first classifies SAE occurrence and subsequently estimates its time of onset. This approach enables modeling of true non-occurrences as well as predicting event onset dates when an SAE is expected. Together, these models can act as proof-of-concept to provide a strategic roadmap for optimizing patient management without compromising safety, which is essential given the increasing strain on hospital resources due to demographic changes and the growing number of MM patients.

## Results

### Patient characteristics

Table 1A summarizes the baseline patient characteristics. In total, 109 patients were included. The median age was 63 years (range 41–79), with 63% being male. The Revised International Staging System (R-ISS) was available for 64 patients: 6 (9%) had stage I, 42 (66%) had stage II, and 16 (25%) had stage III MM. IgG kappa (43; 39%) was the most common myeloma subtype, followed by IgG lambda (27; 23%) and IgA kappa (10; 9%). The most frequent types of induction therapy regimens were DVTd (daratumumab, bortezomib, thalidomide, dexamethasone) in 45 (41%) patients and VCD (bortezomib, cyclophosphamide, dexamethasone) in 41 (38%) patients. Serologic response status upon SCM was available for 89 patients: 44 (49%) were in VgPR, 40 (45%) in PR, 4 (5%) were in SD and 1 (1%) in PD (see Table 1A).

**Table 1:** Overview of cohort (*N* = 109) characteristics

A—Patient characteristics at diagnosis	*N* = 109 (%)
Median age, years [min, max]	63 [41, 79]
Sex (M/F)	69 (63)/40 (37)
Stage R-ISS (available for 64 pats.):	
• I	6 (9)
• II	42 (66)
• III	16 (25)
Median plasma cell infiltration of bone marrow at diagnosis (available for 87 pats.):	
• 10–29%	16 (19)
• 30–59%	29 (33)
• 60–100%	42 (48)
Heavy chain type and sFLC:	(kappa/lambda/none)
• IgA	10/5/2 (9, 5, 2)
• IgG	43/27/1 (39, 25,1)
• IgM	0/1/0 (0, 1, 0)
• Bence Jones	2/2/0 (2, 2, 0)
• None	8/6/2 (7, 5, 2)
Pretreatment (multiple possible):	
• DVTd	45 (41)
• VCd	41 (38)
• Vd	8 (7)
• EKRd	5 (4.6)
• VTd	5 (4.6)
• VRd	5 (4.6)
Response status before collection (available for 89 pats.):	
• VgPR	44 (49)
• PR	40 (45)
• SD	4 (5)
• PD	1 (1)

B—Therapy, time schedule, and outcome	
Successful stem cell collection:	106 (97)
Duration between admission and therapy start (days):	
• 0–1	101 (93)
• 2–4	8 (7)
Chemotherapy regime *n* (%):	
• Etoposide 375 mg/m^2^	10 (9)
• Etoposide and cyclophosphamide 100 mg/m^2^/1250 mg/m^2^	44 (40)
• Cyclophosphamide 2500 mg/m^2^	55 (51)
Leukopenia start after start of therapy (days):	
• 6–8	91 (84)
• ≥9	9 (8)
• None	9 (8)
Median: 7 (0.25 and 0.75 percentile: 7 and 8) days	
Duration of Leukopenia (available for 100 patients) (days):	
• 1–3	33 (33)
• 4–5	52 (52)
• 6–8	15 (15)
Median: 4 [0.25 and 0.75 percentile: 3 and 5] days	
Time between the start of therapy and stem cell collection (days):	
• 10–12	36 (34)
• 13–15	57 (51)
• 16–19	13 (12)
• Not successful	3 (3)
Median: 13 [0.25 and 0.75 percentile: 12 and 14] days	
Days of stem cell collection (available for 106 patients) (days):	
• 1	77 (73)
• 2	27 (25)
• 3	2 (2)

C—Adverse events	
Adverse event (clinically documented)	81 (74)
Severe adverse event	75 (69)
Adverse event (CTCAE ≥ 3)	65 (60)
• Nausea	21 (19)
• Diarrhea	5 (4.5)
Median start of symptoms [range 1–16] days	4 [days]
• Neutropenic fever	59 (54)
• Germ detection	26 (44)
Median start of neutropenic fever [range 1–12] days	9 [days]
• Erythrocyte transfusion	28 (26)
Median administration of red cell concentrates [range 9–14] days	11 [days]
• Mild renal impairment	12 (11)
Median Start of mild renal impairment [range 1–14] days	11 [days]
• Acute kidney injury (AKI)	
AKI-I	8 (7)
AKI-II	0 (0)
AKI-III	0 (0)

Crude rates or median times and ranges or percentiles were only calculated on the subset of patients developing the respective adverse event. Severe adverse events are defined as adverse events requiring hospitalization, including due to fever (≥38.2°C body temperature), mild renal impairment (≥1.2 mg/dl plasma creatinine), administration of antibiotics, transfusion of platelets or erythrocytes.

Other adverse events included nausea and diarrhea.

*M* male, *F* female, *R-ISS* revised international staging system, *sFLC* serum free light chains, *Ig* immunoglobulin A, G, M, *VCd* bortezomib, cyclophosphamide, dexamethasone, *DVTd* daratumumab, bortezomib, thalidomide, dexamethasone, *VTd* bortezomib, thalidomide, dexamethasone, *EKRd* elotuzumab, carfilzomib, lenalidomide, dexamethasone, *VRd* bortezomib, lenalidomide, dexamethasone, *Vd* bortezomib, dexamethasone, *VgPR* very good partial response, *PR* partial response, *SD* stable disease, *PD* progressive disease, *pats* patients, *AKI* acute kidney injury, *CTCAE* Common Terminology Criteria for Adverse Events.

### Therapy time schedule and outcome

**Fig. 1:**
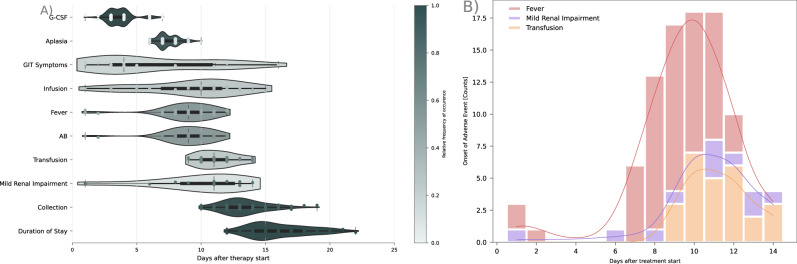
Temporal profile and distribution of adverse event occurrences. **A** Overview of the temporal relationships and distributions of stem cell collection milestones and adverse events. Each dot marks the first occurrence or administration of a variable, with small vertical lines indicating the first and last event observations. Color intensity reflects the proportion of patients experiencing the event, with darker colors representing higher fractions. Density is estimated via kernel density estimation (KDE). **B** Distribution estimates of the first occurrence of severe adverse events (SAEs), which would normally result in hospitalization. A stacked histogram and KDE, highlighting the bimodal distribution between patients who develop an SAE within 72 h and those who do so later. Mild renal impairment: >1.2 mg/dL creatinine; G-CSF Granulocyte Colony Stimulating Factor, AB Antibiotic Therapy.

Stem cell mobilization regimens, timing and outcomes are summarized in Table 1B and Fig. 1A. Chemotherapy was initiated within one day of admission in 101 (93%) patients. Cyclophosphamide (4 g/m^2^ over 2 days) was administered in 48 (44%) patients, while 44 (40%) received etoposide (100 mg/m^2^) plus cyclophosphamide (2500 mg/m^2^) over 3 days. Ten patients (9%) received etoposide alone (500 mg/m^2^) over 4 days. Leukopenia occurred in 100 patients (92%), with a median onset on day 7 (range 6–10) and a median duration of four days (range 1–8). The median interval from therapy initiation to stem cell collection was 13 days (range 10–19). Overall, 106 patients underwent successful stem cell collection, typically over the course of one (73; 73%) or two days (27; 25%).

### Adverse events after mobilization chemotherapy

Table 1C and Fig. 1A summarize adverse events (AEs) over time. Overall, 75 patients (69%) experienced at least one clinically relevant AE, defined as symptomatic or intervention-requiring events. Among these, 65 patients (60%) developed at least one AE defined as CTCAE grade ≥3 or requiring hospitalization. Nausea (*n* = 21) and diarrhea (*n* = 5) were clinically documented in 24 (22%) patients after a median of 4 days (range 1–16). 59 patients (54%) developed neutropenic fever at a median of day 9 (range 1–12), necessitating antibiotic therapy in all cases. Elevated creatinine levels ( ≥ 1.2 mg/dl) occurred in 12 patients (11%) at a median of day 10 (range 1–14) and did prompt supportive treatment with IV fluids. Upon admission, one patient had acute kidney injury (stage 3^10^) and ten had pre-existing chronic kidney disease (G3a: 6, G3b: 2, G4: 2; all A1^10^). Notably, none of the patients with impaired renal function experienced worsening of CKD stage by discharge, nor did they have significantly lower stem cell yields or increased collection failures. Anemia manifested in 28 patients (26%), with a median onset on day 11 (range 9–14), prompting erythrocyte transfusions. Side effects occurring within the first 72 h were clearly separated from those arising at later times (Fig. 1B). Neutropenic fever was the main cause for hospitalization (Fig. 1C).

Subgroup analysis of patients who received 4 mg/m^2^ cyclophosphamide and/or had received prior DVTd therapy showed no significant effect (individually or interacting) on stem cell yield, timing of collection start or incidence or onset of SAEs (see Supplementary Tables 2-5).

### Outpatient modeling

**Fig. 2:**
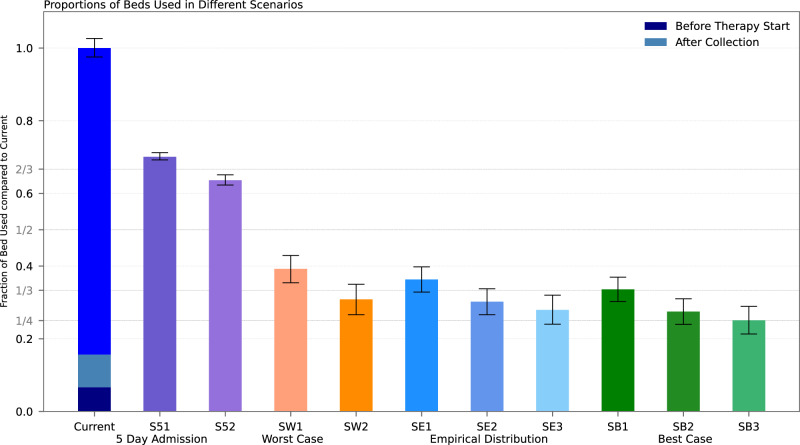
Estimated number of needed hospital beds per day for simulated outpatient regimes. The relative fraction on needed bed-days is given. Error bars are bootstrapped 95%-CI. For scenarios ending on a 2, the therapy administering was also ambulant. For scenarios ending on 3, the SCC was also in an outpatient regime, if no prior SAEs occurred. *Current:* current full inpatient treatment; set as normalizing constant for relative fractions. Additionally, the fractions of needed bed-days between admission and therapy start as well as the occupied bed-days after SCC end are given. *5 Day Admission:* all patients not developing SAEs within the first 72 h were admitted to the hospital 5 days after therapy start. *Worst case*: all patients developing NF did this one day earlier. *Empirical distribution:* following the observed SAE distributions. Best Case: all patients developing NF did this one day later. SAE severe adverse events, NF neutropenic fever, SCC stem cell collection.

Figure 2 illustrates the estimated hospital bed requirements per day (bd) under different outpatient treatment strategies using our modeling. In all scenarios, the number of required beds per day (bd) is significantly lower than that of the current inpatient regimen, including the delayed admission until day 5 (*p* = 3.1 × 10^−^^35^) and worst-case scenarios (*p* = 9.9 × 10^−39^). Under the current inpatient approach, a total of 1754 bd were occupied, of which 117 bd (6.7%) were needed between hospital admission and therapy initialization and 157 bd (9%) after stem cell collection. Patients experiencing early SAEs (within 72 h) occupied 66 bd (3.8%), which corresponds to their proportion in the cohort (3.6%, 4 out of 109 patients). Despite the small sample size, these patients did not have a significantly longer stay than the rest of the cohort (*p* = 0.72).

In the “cautious” scenarios, when patients without early SAE are admitted on day 5, total bed-day demand decreases by 30–36%. The worst-case scenarios still achieved a reduction by 61–66%. Realistic outpatient strategies reduced bed-days by 64–73%. Best-case scenarios decreased bed-days by 67–75%, lowering total bed-days to as few as 440 versus 1754 under the current regimen. Additional simulation further shifting towards outpatient regimens for the treatment of mild renal (serum creatinine >1.2 mg/dL) impairment, blood transfusion and/or neutropenic fever reduces needed bed-days drastically (see Supplementary Fig. 1 and Supplementary Table 8**)**.

### Predictive modeling SAEs

The predictive models demonstrated varying performance depending on the type of SAE. For elevated creatinine levels, the Random Forest (RF) classifier achieved the highest accuracy (0.96), while XGBoost attained a perfect ROC-AUC (1.00) and a strong Matthews correlation coefficient (MCC) of 0.78. In predicting the need for blood transfusions, Random Forest again performed well regarding an accuracy of 0.90 and a ROC-AUC of 0.91, whereas TabPFN demonstrated the highest MCC (0.48). For neutropenic fever prediction Gradient Boosting yielded the highest accuracy (0.90), but Logistic Regression provided the best MCC (0.40) and the highest ROC-AUC (0.67). Overall, for predicting the occurrence of any SAE, XGBoost achieved the highest accuracy (0.79), Logistic Regression yielded the highest ROC-AUC (0.81) and TabPFN the highest MCC (0.48). See also Table 2. For the regression of the onset of first SAE, the following average errors are reported: Elastic Net: 1.65 days (d) mean absolute deviation (MAD), 2.04 d root mean squared deviation (RMSD); Random Forest Regressor: 1.42 d MAD, 1.79 RMSD; TabPFN-Regressor: 1.45 d MAD, 1.75 RMSD, XGBoost-Regressor: 1.33 d MAD, 1.66 d RMSD. See Supplementary Tables 6 and 7 for additional model descriptions. Predicting SAEs within the first 72 h was unfeasible given only four events.

**Table 2:** Mean Scores for the best performing classifier in a 5-fold cross-validation of our dataset

Target	Metric	Classifier	Mean score
MRI			
	Accuracy	Random Forest Classifier	0.96
	ROC-AUC	XGBoost Classifier	1.0
	MCC	XGBoost Classifier	0.78
Fever			
	Accuracy	Gradient Boost Classifier	0.9
	ROC-AUC	Logistic Regression	0.67
	MCC	Logistic Regression	0.4
Transfusion			
	Accuracy	Random Forest Classifier	0.9
	ROC-AUC	Random Forest Classifier	0.91
	MCC	TabPFN Classifier	0.48
Any AE			
	Accuracy	XGBoost Classifier	0.79
	ROC-AUC	Logistic Regression	0.81
	MCC	XGBoost Classifier	0.52

MRI: prediction of supportive IV Fluid due to mild renal impairment; fever: neutropenic fever; transfusion needs; any AE: composite Endpoint of occurrence of any of the above adverse events.

Hyperparameter-tuning was performed within nested cross-validation.

## Discussion

Based on our inpatient setting, our analysis provides strong evidence that, at least partially, outpatient stem cell mobilization in multiple myeloma is both safe and feasible. Our simulations suggest that adapted patient admission policies could even in conservative scenarios reduce bed occupancy up to 75% compared to the current standard inpatient treatment (see Fig. 2). By additionally treating selected adverse events in an outpatient setting, hospital bed occupation could be reduced by more than 90% of bed-days overall (see Supplementary Fig. 1). This approach is particularly effective when combined with personalized ward bed management strategies based on predicted hospital admissions, for which we propose a two-step framework to predict (1) whether and (2) when adverse events might occur.

Unlike previous studies that either excluded anti-CD38-based induction or evaluated only low/intermediate-dose mobilization regimens^11–13^, our study assesses current protocols – including CD38-containing induction therapies and high-dose mobilization with cyclophosphamide (4 g/m²) and etoposide^9^. Using these protocols, we found no increase in SAE rate, which is consistent with existing literature^14,15^. This is expected, as outpatient mobilization with antibiotic prophylaxis is already practiced in parts of the European Union (e.g., Italy) and also in the United States—with similar reported SAE rates^11–13^. Nevertheless, we anticipate that a small fraction of patients (approximately 3–5%, as observed) will remain ineligible for outpatient SCM, particularly those developing early SAEs. The observed temporal dynamics suggest that these events stem primarily from patient-specific factors rather than the mobilization chemotherapy itself, as affected individuals presented with compromised clinical status (e.g., pre-existing acute kidney injury) at admission.

Beyond our described use case, the two-step prediction framework (first predicting if, then when an adverse event occurs) can be adapted to virtually every other situation where a genuine non-occurrence probability exists rather than mere right-censoring. Classical survival models assume that an event will eventually occur if waited long enough; this does not hold for short-term adverse events, where a non-zero subset of patients may never develop an adverse event. The two-step approach mitigates this and performs better than the survival and cure model approaches we tested. An additional benefit is that the first classifier can be tuned according to the severity of a potential misclassification, thereby reducing potential harm in outpatient settings.

As febrile neutropenia (FN) was the primary cause of hospitalization, accounting for 59 of the 71 initial SAEs, preventing FN seems critical to reducing inpatient stays. However, the literature reports mixed findings regarding FN incidence in outpatient settings^16,17^. Our models capture this uncertainty through best- and worst-case scenario simulations. Furthermore, recent studies indicate that selected low-risk FN cases can be managed safely in outpatient settings^18–20^. A stringent risk stratification, therefore, allows for further reductions in bed requirements, as reflected in our supplementary simulations.

Beyond infection management, regular laboratory monitoring remains essential in outpatient settings to enable early intervention and prevent or promptly detect the need for subsequent hospitalization. Our supplementary models account for this by incorporating the outpatient treatment of specific adverse events (see Supplementary Fig. 1). For instance, we classified mild renal impairment, as indicated by serum creatinine >1.2 mg/dL, as a specific trigger for proactive supportive care (e.g., intravenous fluids), given that early treatment is critical for favorable outcomes^21^. While our models accurately predicted renal decline, often preceded by gradual changes, transfusion requirements proved less predictable. Consequently, we recommend closer monitoring for patients with pre-existing renal dysfunction or cytopenia in any hematologic lineage.

Although our single-center cohort may be representative of different clinics as well, generalizability requires confirmation in multicenter settings. To facilitate reproducibility and external evaluation, we provide the full covariate set and final hyperparameters in Supplementary Tables 6 and 7, enabling straightforward implementation and testing across centers. Furthermore, we limited our models to routinely available laboratory values present in most centers and outpatient settings at fixed time points. This strategy ensures consistent model input and prevents information leakage from selectively ordered tests that reflect underlying pathological conditions. To account for different institutional protocols regarding the management of specific adverse events, e.g., whether to give transfusions in an in- or outpatient setting, we added additional simulations, providing initial assessments for a diverse range of clinical pathways. Furthermore, as no standardized guidelines exist, physicians may have selected less intensive regimens for frailer patients, potentially biasing the observed adverse event rates themselves. Exploratory subgroup analyses indicate potential differences between treatment regimens; however, this needs to be validated in adequately powered studies. Sensitivity analyses show substantial improvement with larger patient subsamples, suggesting that model performance would substantially benefit from additional patients. Nevertheless, our final models demonstrate good accuracy and ROC-AUC performance. To more reliably convey clinical utility—especially in the presence of class imbalance—we also report the more stringent MCC. Interestingly, TabPFN^22^, a new foundation model for tabular data, performed consistently well but did not outperform the other methods as one might have expected.

Additional predictors, such as body temperature (unavailable due to a lack of digitalization) or plasmacytosis/ bone marrow staging before SCC, might also prove beneficial. The latter was only available upon initial diagnosis and was not routinely performed right before SCM due to limited therapeutic consequences. Additionally, sparse data for later timepoints and inconsistent laboratory sampling prevented a meaningful evaluation of short-term SAE forecast quality.

Although chemotherapy-based mobilization regimens are the standard for eligible patients, steady-state mobilization strategies with reduced toxicity, such as G-CSF plus plerixafor, exist^23^. However, their use remains limited due to cost, reimbursement constraints, institutional policies, and reduced mobilization efficiency^24^. As this work is designed to fit within current clinical pathways and can be implemented immediately, it offers resource optimization even before broader transitions to plerixafor-based strategies might occur.

Due to differing toxicity profiles, these chemotherapy-based findings should not be extrapolated to steady-state mobilization.

The data show a strict bimodal distribution of SAE occurrences (before 72 h or after 5 days), indicating a small but robust window for a safe, minimal outpatient regimen. The admission on day 5 scenario is therefore directly implementable with minimal changes in clinical workflows, still having high practical relevance, as approximately one-third of required ward bed-days could be saved. An initial transition to that scenario also gives time to establish suitable outpatient infrastructures and medical networks, including primary-care support with accessible diagnostics and specialized staff, that are necessary for extended outpatient workflow. In a later stage, this fixed admission policy should be refined with personalized admission estimates. For maximal efficiency, prediction errors should be less than one day. Unfortunately, our regression models, trained only on admission-day data, were slightly higher than this threshold. However, future incorporations of longitudinal models or conditional predictions will likely improve model performance.

A formal cost-effectiveness analysis was not performed, though economic benefits are implied by the substantial reduction in bed-days. Furthermore, the economic impact will depend heavily on country-specific reimbursement policies. Regardless of economic considerations, transitioning to outpatient settings addresses an immediate patient need, as current studies consistently show a strong preference among cancer patients for the quality-of-life benefits associated with ambulatory care^25–27^. This preference likely extends to stem cell mobilization in multiple myeloma patients^14,28,29^, providing a compelling motivation to utilize current protocols immediately rather than waiting for future mobilization strategies to mature.

**Fig. 3:**
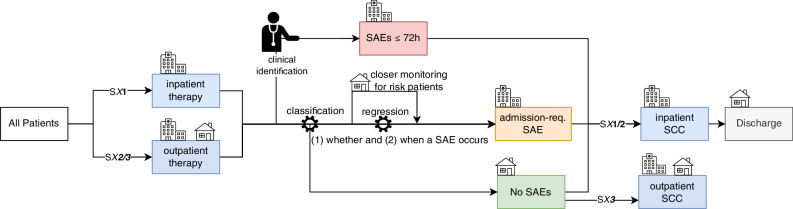
Overview of the proposed model structure for risk-stratified treatment regimens for patients with multiple myeloma undergoing stem-cell mobilization and collection (SCC). Early severe adverse events (SAEs) occurring within the first 72 h of hospital admission are usually clinically identifiable. The remaining patients are screened for the probability of developing an SAE requiring hospitalization. After an initial classification, subsequent regression algorithms predict the day of SAE onset for relevant patients, supporting efficient ward-bed management. SX1, SX2, and SX3 denote different sub-model assumptions: SX1: therapy administration and SCC performed in an inpatient setting; SX2: therapy administration on an outpatient basis, SCC in an inpatient setting; SX3: therapy administration and SCC on an outpatient basis if the patient has not required hospitalization before SCC.

In summary, our analysis strongly suggests that outpatient stem cell mobilization in multiple myeloma is both safe and feasible, with stratified admission policies and optimized bed management markedly reducing inpatient bed usage—potentially by at least one-third and up to more than 90%. Predictive modeling of SAEs remains challenging due to limited sample size, data availability, and class imbalance, but longitudinal approaches and conditional prediction with small event horizons show promise for future refinement. Finally, the general structure of the proposed model (Fig. 3) can serve as a blueprint for other high-risk treatment pathways in multiple myeloma, particularly bispecific antibodies and CAR T-cell therapy, as well as for other disease entities or further extensions and refinements, for example, by integrating more performant models in settings with greater data availability.

## Methods

We retrospectively analyzed 109 cases of adult patients with MM who underwent first-line chemotherapy induction for SCM with subsequent high-dose chemotherapy and ASCT in an inpatient setting at the Department of Hematology and Medical Oncology, University Medical Center Göttingen (UMG), Germany, between August 2019 and December 2022. MM diagnosis was confirmed by local pathologists according to international criteria^30^. All patients had an Eastern Cooperative Oncology Group (ECOG)^31^ performance status of 0–1 and consented to undergo stem cell mobilization. All response statuses and chemotherapy regimens were included. Mobilization chemotherapy consisted of either cyclophosphamide 4000 mg/m^2^ or cyclophosphamide 2500 mg/m^2^, etoposide 375 mg/m^2^ or etoposide 100 mg/m^2^ plus cyclophosphamide 1250 mg/m^2^ (see Table 1B). G-CSF (10 µg/kg/day subcutaneously) was administered following chemotherapy. Clinical data were extracted from medical records and electronic patient files supplemented by additional patient-related documents. Some baseline assessments were conducted off-site, and the associated data were not reliably available to our center in retrospect. The study was approved by the UMG Ethics Committee (Goettingen no. 24/1/23) and conducted in accordance with the Declaration of Helsinki, Good Clinical Practice guidelines and local regulations.

### Response assessment and adverse event grading

Remission status prior to mobilization was classified according to the International Myeloma Working Group (IMWG) uniform response criteria as very good partial response (VgPR), partial response (PR), stable disease (SD), or progressive disease (PD)^32^. VgPR was assessed per IMWG criteria using serum M-protein and serum free light-chain measurements. In line with German clinical practice, bone marrow aspiration/biopsy was not routinely performed immediately before SCM. All clinically relevant adverse events during mobilization chemotherapy were graded according to the Common Terminology Criteria for Adverse Events (CTCAE) Version 5.0^33^. Asymptomatic laboratory abnormalities that did not require clinical evaluation or intervention were not recorded as adverse events. Day 0 was set to therapy start. Severe adverse events (SAE) were defined as CTCAE grade ≥ 3, requiring in-hospital management. Mild renal impairment was defined as a serum creatinine concentration >1.2 mg/dL, irrespective of baseline. This threshold was chosen pragmatically to capture subclinical renal dysfunction not meeting CTCAE grade 3 or Kidney Disease Improving Global Outcomes (KDIGO) criteria but triggering clinical intervention (IV fluids). Acute kidney injury (AKI) was defined according to KDIGO guidelines, including an increase in serum creatinine by ≥0.3 mg/dL within 48 h, a ≥ 1.5-fold increase from baseline within 7 days, or urine output <0.5 mL/kg/h for >6 h.

Leukopenia was defined as an absolute leukocyte count <1000/µl. Side effects included nausea or diarrhea, fever (>38.2 °C), infection and anemia or thrombocytopenia requiring transfusion. Patients with fever were also classified as having an infection with and without germ detection, and antibiotic therapy was documented. Transfusion applications were recorded.

### Statistical analysis and modeling of potential outpatient regimen scenarios

Descriptive statistics, including a chronological summary of adverse events, were performed to characterize cohorts. Subgroup analysis of high-dose cyclophosphamide therapy and DVTd pre-therapy was performed using two-way ANOVA (see Supplementary Tables 2–5).

All patients were admitted before chemotherapy and remained hospitalized until stem-cell collection was completed sufficiently. Based on our inpatient cohort, we estimated empirical distributions of SAE occurrence. To assess alternative admission strategies, we modified these distributions according to each scenario. For each scenario, 95% confidence intervals were calculated using 2000 bootstrap samples. Patients were assigned to three subgroups: (1) those with severe adverse events (CTCAE grade ≥ 3) within 72 h of initiation of chemotherapy, (2) those with SAEs before stem cell collection (“later acquired”) and (3) those without SAEs (see Fig. 3). The proportion of early SAE patients (CTCAE grade ≥ 3 within 72 h) as well as the quota of patients with acute kidney injury (AKI) and transfusions was kept constant in all main simulations. In most scenarios, the onset (not the relative rate) of neutropenic fever as the main cause for hospitalization was varied according to the model’s assumption. The empirical scenarios (SE1/2/3) were based on the observed (inpatient) SAE distributions without any changes. In the worst-case scenarios (SW1/2), fever occurred one day earlier for febrile patients, whereas in the best-case scenarios (SB1/2/3) it occurred one day later. For all patients not developing SAEs within the first 72 h, a fixed admission day on the fifth day after start of therapy was implemented (“Day 5”) scenarios (S51/2). In all other scenarios, patients were immediately hospitalized whenever an SAE occurred.

For further refinement, additional sub-scenarios were developed based on whether patients were hospitalized during therapy application and SCC. In sub-models SX1, therapy was administered in an inpatient setting, whereas in sub-models SX2, therapy was administered in an outpatient setting. In both sub-models (SX1/2), the SCC was performed in an inpatient setting, regardless of whether an SAE occurred. In sub-models SX3, both therapy administration and stem cell collection (if no SAE occurred) were performed in an outpatient setting. See Supplementary Table 1 for a detailed description. The number of ward beds per day needed in each scenario was normalized against the full inpatient (observed) bed-days.

To support optimal ward bed management, a two-step machine learning prediction strategy is proposed (see Fig. 3): first, predicting whether a patient will develop a relevant adverse event, and secondly, when. To predict the occurrence of SAEs that occurred after 72 h of admission, we compared two data input strategies: for one, only information available at hospital admission was used. For the second longitudinal data, including blood laboratory results collected on days 3 and 5 after therapy start, were integrated. Missing covariates (<0.5%) were imputed via running average for repeated measurements or by the population mean otherwise. No outcome data were missing. For the general SAE prediction, the composite endpoint “any SAE” was used, along with three specific SAEs endpoints: mild renal impairment, neutropenic fever (NF) and blood transfusion requirement. Class imbalances range from moderate to severe (compare Table 1C). Whenever possible, weighted training was used to encounter these imbalances. Input variable selection was based on clinical knowledge and high mutual information with the target.

The models were trained using three progressively expansive covariate sets. First, we employed a clinically informed panel encompassing patient age, estimated glomerular filtration rate (CKD-EPI 2021), lactate dehydrogenase, hemoglobin, platelet count, prior cyclophosphamide-etoposide therapy, and the total number of previous treatment lines. Next, we selected features by their mutual information with the outcome, extending the first set to include patient sex, C-reactive protein, corrected calcium, serum creatinine, chemotherapy dose indicator (full dose = 1), and binary flags for cyclophosphamide, CE-therapy, etoposide, and G-CSF dosing. Finally, we augmented this mutual-information set with longitudinal measurements, adding all blood values recorded on days three and five after treatment initiation.

We evaluated four machine-learning classifiers, including Logistic Regression, Random Forest (RF), XGBoost^34^, and TabPFN^22^. The primary classification task was to predict whether a patient would develop a specific SAE. For patients who did develop a SAE, regression models were used to estimate the timing of the first SAE occurrence. We used four regression models: a linear regressor (Elastic Net)^35^, an XGBoost regressor^34^, a TabPFN regressor^22^, and an RF regressor^35^. For the regression task, we used only features collected on the day of admission. Given the limited sample size, we performed 5-fold cross-validation (CV) for classification and 3-fold CV for regression to ensure reliable performance evaluation. For the classification tasks, results are reported as average across all five validation folds for accuracy, ROC-AUC, and the MCC^36^, and the RMSD and the MAD for all regression tasks. We report the mean over all CV-folds. Hyperparameters were tuned manually in a nested CV framework. All statistical analyses and visualizations were conducted using Python 3.10.12^37^, employing open-source libraries^22^^,^^34^^,^^35^^,^^37–39^, and custom scripts. See Supplementary Tables 6 and 7 for additional descriptions and Supplementary Table 9 for a complete TRIPOD + AI^40^ summary of the used models.

## Supplementary information

Supplementary information

## Data Availability

The datasets generated and/or analyzed during the current study are not publicly available due to privacy and ethical restrictions, but may be available from the corresponding author upon reasonable request.

## Code availability

All scripts used for the analysis will be available upon reasonable request.
