# Supplementary material for: Predicting adverse events for risk stratification of chemotherapy based stem cell mobilization in multiple myeloma
Source: NPJ Digit Med. 2026 Feb 3;9:203. doi: 10.1038/s41746-026-02394-y (PMC12966404; doi:10.1038/s41746-026-02394-y)
Supplement: Supplementary file 1 — Supplementary information [file 41746_2026_2394_MOESM1_ESM.pdf]

Supplement 1 – Main Simulations

Model	Description	Needed Bed Days	Reduction In %
Current	Summation of observed bed usage.	1754	0
SE1	Empirical Model; hospitalisation when a SAE occurs; inpatient therapy administration and collection	637	64%
SE2	Empirical Model; hospitalisation when a SAE occurs; outpatien therapy administration; inpatient collection	530	70%
SE3	Empirical Model; hospitalisation when a SAE occurs; outpatient therapy administration and collection, if the patient did not develop any SAE before collection	490	72%
SW1	Worst case model; in patients with FN, fever occurs one day earlier; hospitalisation when SAE occurs; inpatient therapy administration and collection	688	61%
SW2	Worst case model; in patients with FN, fever occurs one day earlier; hospitalisation when SAE occurs; outpatient therapy administration and inpatient collection	541	69%
SB1	Best case model; in patients with FN experience fever one day later; hospitalisation when SAE occurs; inpatient therapy administration and collection	589	66%
SB2	Best case model; in patients with FN experience fever one day later; hospitalisation when SAE occurs; outpatient therapy administration and inpatient collection	482	73%
SB3	Best case model; in patients with FN experience fever one day later; hospitalisation when SAE occurs; outpatient therapy administration and collection, if the patient did not develop any SAE before collection	440	75%
S51	Cautious model: Patients with SAEs occurring after 72 hours are admitted to the hospital on day 5 after therapy, regardless of whether they have SAEs; inpatient therapy administration and collection	1229	30%
S52	Cautious model: Patients with SAEs occurring after 72 hours are admitted to the hospital on day 5 after therapy, regardless of whether they have SAEs; outpatient therapy administration and inpatient collection	1116	36%

Supplementary Table 1: Detailed description for all simulation scenarios. For all models: No changes were made for patients developing early SAEs (<72h); the distributions of mild renal impairment (>1.2mg/dL creatinine) and transfusions were also not changed. SX1: therapy administration and SCC performed in an inpatient setting; SX2: therapy administration on an outpatient basis, SCC in an inpatient setting; SX3: therapy administration and SCC on an outpatient basis if the patient has not required hospitalization before SCC.
SAE: severe adverse event; FN: febrile neutropenia; SCC: stem cell collection.

Supplement 2: Subgroup Analysis

Naïve subgroup analysis was performed to determine whether more modern induction regimens (e.g., including pre-therapy with DVTd) and intermediate-to-high cyclophosphamide doses (up to 4 g/m^2) differed significantly from the rest of our cohort. We evaluated whether prior DVTd induction and intermediate-to-high cyclophosphamide dose were associated with: (1) weight-adjusted stem cell yield ($\times 10^6$ $CD34^+$ cells/kg), (2) day of first stem-cell collection after therapy start, (3) occurrence of any relevant adverse event (composite), and (4) onset day of the first relevant adverse event among patients with an event. Relevant AEs were defined as neutropenic fever, administration of IV fluids for decreased renal function, or blood transfusion for anemia. Analyses were exploratory; p-values are two-sided and unadjusted for multiplicity.

All models were fit using Python (v3.12.9) and the statsmodels library (v0.14.4). For the binary outcome (occurrence of any relevant AE) we fitted a logistic regression model and conducted an analysis of deviance. For all other outcomes, we used simple linear regression followed by ANOVA.

No significant differences can be found between the subgroups.

---

(1) Number of Collected Stem Cells

Neither the omnibus F-test ($F(3, 105) = 2.172$, $p = 0.0957$) nor any individual term reached significance, indicating that neither prior DVTd therapy, intermediate-to-high dose cyclophosphamide, nor their interaction significantly affected the number of stem cells collected (N = 109).

Source	df	sum_sq	mean_sq	F	PR(>F)	partial η^2
DVTd	1	2.8750	2.8750	2.9414	0.0893	0.0273
Cyclo	1	2.5969	2.5969	2.6568	0.1061	0.0247
DVTd × Cyclo	1	0.8967	0.8967	0.9174	0.3404	0.0087
Residual	105	102.6313	0.9774	—	—	—

Supplementary Table 2: ANOVA: Number of collected stem cells. See text above for further descriptions.

(2) Day of Stem Cell Collection after Therapy Start

Neither the omnibus F-test ($F(3, 102) = 1.390$, $p = 0.250$) nor any individual term reached significance, indicating that neither prior DVTd therapy, intermediate-to-high dose cyclophosphamide, nor their interaction significantly affected the number of collected stem cells (N = 106).

Source	df	sum_sq	mean_sq	F	PR(>F)	partial η²
DVTd	1	1.283630	1.283630	1.285697	0.259500	0.012448
Cyclo	1	1.348646	1.348646	1.350817	0.247848	0.013070
DVTd × Cyclo	1	1.531643	1.531643	1.534108	0.218340	0.014817
Residual	102	101.836080	0.998393	—	—	—

Supplementary Table 3: ANOVA: Day of first stem-cell collection after therapy start. See text above for further descriptions.

(3) Occurrence of Any Relevant Adverse Event

Neither the omnibus likelihood-ratio test ($\chi^2(3)=3.826$, $p=0.2808$) nor the sequential deviance tests for main effects ($\chi^2(2)=1.925$, $p=0.3820$) or interaction ($\chi^2(1)=1.902$, $p=0.1679$) reached significance, indicating that neither prior DVTd therapy, intermediate-to-high cyclophosphamide dosing, nor their interaction significantly affected SAE (N=109).

Source	df	LR Stat	p-value
Omnibus (full vs null)	3	3.826	0.2808
Main effects vs null	2	1.925	0.3820
Interaction vs main	1	1.902	0.1679
Residual	105	–	–

Supplementary Table 4: Logistic regression (analysis of deviance): Occurrence of any relevant adverse event. See text above for further descriptions.

(4) Onset of First Relevant Adverse Event

Neither the omnibus F-test ($F(3, 68) = 1.001$, $p = 0.398$) nor any individual term reached significance, indicating that neither prior DVTd therapy, intermediate-to-high dose cyclophosphamide, nor their interaction significantly affected the onset of the first adverse event (N=72).

Source	df	sum_sq	mean_sq	F	PR(>F)	partial η²
DVTd	1	0.342389	0.342389	0.337648	0.563111	0.004941
Cyclo	1	0.206094	0.206094	0.203240	0.653552	0.002980
DVTd × Cyclo	1	2.496570	2.496570	2.461996	0.121272	0.034941
Residual	68	68.954946	1.014043	—	—	—

Supplementary Table 5: ANOVA: Onset day of first relevant adverse event (among patients with an event). See text above for further descriptions.

Supplement 3 – Model Description

Predicting Adverse Events

We evaluated binary classifiers for their respective ability to **classify the occurring of the following adverse events**:

1) Neutropenic Fever Occurrence
2) Requirement for Blood Transfusion
3) Administration of IV fluids
4) Any of the above, as composite endpoint

Four model classes were used:

A) **Logistic Regression** (Sklearn)
B) **Random Forest Classifier** (Sklearn)
C) **XGBoost/Gradient Boost Classifier**
D) **TabPFN Classifier**

Logistic regression served as our linear baseline, while the random forest enabled nonlinear predictions with interpretable feature importances. XGBoost is a powerful, regularized gradient-boosted tree algorithm that routinely ranks among the top-performing models. TabPFN, a newer foundation model, was included to compare its performance in a medical setting, where published results remain limited.
All models are widely used and of increasing model complexity; therefore, they enable finding the smallest model class suitable for prediction and reducing the risk of overfitting. Interestingly, TabPFN did not outperform the other models in our prediction task, as one could have assumed.

The following **covariate sets** were tested for their best suitability:

1. **Clinically informed covariate set**: Age, eGFR (CKD-EPI 2021), LDH (P), Hb, Thrombocytes, pre-therapy with CE; total number of pre-therapies

2. **Selected via highest mutual information**: Age, Sex, Hb, Thrombocytes, CRP (P), Calcium (P, corrected), Creatinine (P), LDH (P), Dose of Chemotherapy (1=full dose), total number of pre-therapies, Cyclo-Therapy (binary), CE-therapy (binary), Eto-therapy (binary), G-CSF (dose)

3. **Longitudinal covariates**: <u>same as 2.</u>, but additionally with blood values at day 3 and 5 after therapy start. Therapy start is defined as day=0.

Models using longitudinal covariates outperformed the other models. The covariate set 2 outperformed the clinically informed covariate set. No single predictor drove performance; incorporating more comprehensive feature sets consistently improved results. Results were reported using covariate set 3 (longitudinal).
Due to the limited number of patients and partly high class imbalances, we decided to use 5-fold cross-validation (CV) as an evaluation framework. As evaluation metrics, we used accuracy, ROC-AUC and Mathew's Correlation Coefficient (the latter two being less sensitive to class imbalance).
Hyperparameter fitting was done in a nested CV framework, in line with current best practices, to avoid potential information leakage from one-fold to others and exposing the full dataset. If standardization of variables was performed, it was only done based on information available within one fold. Sklearn-Pipelines were used, whenever possible.
If possible, we weighted the respective samples inversely to their class frequency, reducing the chance of overfitting in class imbalances. In principle, prediction results can be tuned towards hospitalization to avoid potential harm for missing at-risk patients.

To assess stability, we ran the CVs multiple times with different initial seedings. Results were comparable; we always reported the first (seed) run.

All covariates should be commonly available in nearly all clinical settings. Therefore, similar models should be easily implementable in other centers.

The additional strategies were *exploratorily* deployed but provided inferior results and were not further considered:

- Using Support Vector Classifier for binary outcome prediction
- Dimensionality Reduction (and decorrelation) of input features using PCA.
- Using Cox-Proportional Hazard-Fitter for a time-to-event style prediction.

Final Hyperparameters (if not stated, default values can be assumed):

**Method**	**Hyperparameters**
Logistic Regression	liblinear solver, penalised with l2-norm
RF Classifier	n_est.=100, max_depth=3
Gradient Boost	n_est=100, max_depth=3
XGBoost	n_est=100
TabPFN	softmax=0.95

Supplementary Table 6: Final hyperparameters for binary AE prediction: tasks (NF, transfusion, IV fluids, composite), using the longitudinal covariate set; 5-fold nested CV with class weighting; metric: ROC-AUC.

Regression Model

We then evaluated four **regression models to predict time to adverse event onset** (for patients who experienced the respective event):

5) Neutropenic Fever Occurrence
6) Blood Transfusion Requirement
7) IV Fluids Administration
8) Any of the above, as composite endpoint

Four model classes were used:

E) **Linear Regression (Elastic Net Regularization)**
F) **Random Forest Regressor**
G) **XGBoost Regressor**
H) **TabPFN Regressor**

Only covariate sets 1 and 2 were considered. Because regression applied only to affected patients, the sample size was smaller; thus, we used 3-fold CV with nested hyperparameter tuning on the first fold. We report root mean squared deviation (RMSD) and mean absolute deviation (MAD). Stability was checked via multiple seed runs; results were consistent.

Final Hyperparameters (if not stated, default values can be assumed):

**Model**	**Hyperparameters**
Elastic Net	alpha=0.8, l1 ratio=0.5
RF Regressor	n_est=100, max_depth=5
TabPFN Regressor	default values
XGBoost	n_est=100, learning_rate=0.01, max_depth=15, subsample =0.6, col_sample_bytree=0.6

Supplementary Table 7: Final hyperparameter for time-to-onset regression for AEs: evaluated on the longitudinal covariate set. 3-fold nested CV; metric: MAD.

Supplementary 4: Simulations

***For all models**: In sub-models SX**1**, therapy was administered in an inpatient setting, whereas in sub-models SX**2**, therapy was administered in an outpatient setting. In both sub models (SX1/SX2) the stem cell collection was performed in an inpatient setting. In sub-models SX**3**, both - therapy administration and stem cell collection (if no SAE occurred) - were performed in an outpatient setting*

Overall

Overall, the number of required ward bed days can be further reduced by a fully outpatient regime. Based on our cohort's adverse-event profile, reductions of up to 95% compared to the current situation are possible.

Additional Outpatient Treatment Mild Renal Impairment

This additional model assumes that all patients with mild renal impairment (serum creatinine ≥1.2 mg/dL, irrespective of baseline), who would receive supportive IV fluids in our centre, also receive these infusions but in an entirely outpatient setting. Whereas in the main models this criterion would have necessitated hospitalisation, outpatient administration of fluids can most likely safely occur without patient harm. This assumption does not affect other or subsequent adverse events leading to hospitalisation, which are modelled following the main empirical model (SE). We abbreviate this model as SIV in overview **Supplementary Figure 1**.

Additional Outpatient Treatment Blood Transfusion

This additional model assumes that all patients who would have received a blood transfusion now receive these transfusions in a fully outpatient setting. This administration can likely be done without introducing additional harm to the patient. This assumption does not affect other or subsequent adverse events leading to hospitalisation, which are modelled following the main empirical model (SE). We abbreviate this model as SBT in overview **Supplementary Figure 1**.

Additional Outpatient Treatment Neutropenic Fever

This additional model applies the *MASCC Risk Index for Febrile Neutropenia*[1] to determine whether a patient qualifies as low- or high-risk for poor outcomes when developing neutropenic fever. Low-risk patients may be treated entirely in an outpatient setting. Among patients who developed an initial adverse event after 72 hours, 56 patients subsequently developed neutropenic fever. Of those 56, three would have been classified as high-risk and 23 as low risk, regardless of their inpatient or outpatient status. For the remaining 30 patients, their status at fever onset (inpatient vs. outpatient) would decide whether they were categorized as low or high risk. For modelling purposes, we assumed all 30 were outpatients and thus treated them as such. Consequently, 53 of 56 fever patients could be managed in an outpatient setting. This assumption does not affect other or

subsequent adverse events leading to hospitalization, which are modelled according to the main empirical model (SE). The timing of fever onset for these patients was also simulated from their empirical distribution. We abbreviate this model as SNF in the overview **Supplementary Figure 1**.

Additional Best Case Outpatient Model

This model, combines all outpatient approaches for supplementary IV fluids due to mild renal impairments, blood transfusion and the outpatient treatment of low risk neutropenic fever patients. We abbreviate this model as SAB in overview **Supplementary Figure 1**.
Additional General Worst-Case Scenario (SGWC)
Adverse outcome incidences were analysed within competing risks using the cumulative incidence function estimated via the Aalen–Johansen estimator (AJE)[2,3] (see **Supplementary Figure 2**).
In this supplementary model the cumulative incidence function of the overall first SAE is moved towards the left (by one day). This is a “general worst-case scenario” and assume that all potential adverse events might occur earlier. This model respects the interdependence of the respective adverse events. These models are called SGWC in the overview **Supplementary Figure 1**.

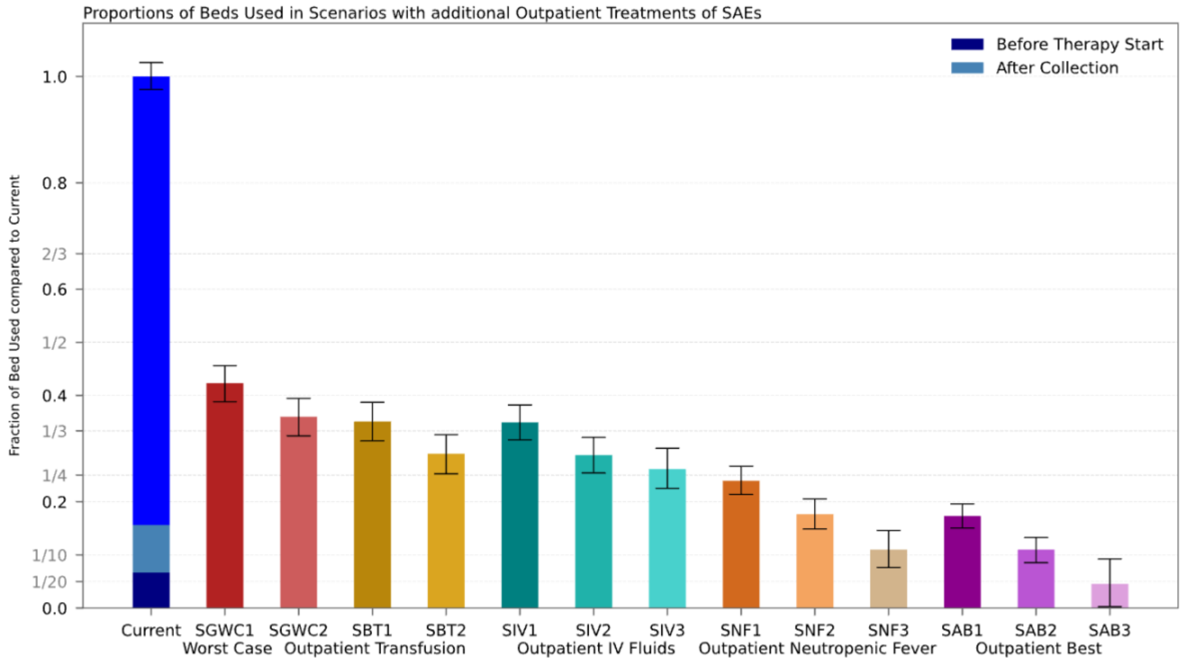

Supplementary Figure 1: Estimated number of needed hospital beds for simulated outpatient regimes. The relative fraction on needed bed-days is given. Error bars are bootstrapped 95%-CI. For scenarios ending on a 1, the therapy administering and SCC is performed in an inpatient setting. For scenarios ending on a 2, therapy was administered on an outpatient basis. For scenarios ending on 3, the SCC was also in an outpatient regime, if no prior SAEs occurred. **Current**: Current full inpatient treatment; set as normalizing constant for relative fractions. Additionally, the fractions of needed bed between admission and therapy start as well as the occupied beds after SCC end are given. **Worst Case**: The overall first SAE occurs one day earlier. **Outpatient IV Fluids**: Supportive IV fluids for mild renal impairment are given in an outpatient setting; rest empirically. **Outpatient Neutropenic Fever**: low risk patients according to the MASCC index are treated in an outpatient

regime. **Outpatient Best**: combines all possible outpatient regimes. For more information see text below. SCC: stem cell collection; SAE: severe adverse event; CI: Confidence Interval.

Model	Bed Day	CI Interval	Reduction (%)
Current	1754	1711–1800	0%
SGWC1	742	681–800	57.7%
SGWC2	631	568–692	64.0%
SBT1	616	552–679	64.9%
SBT2	509	443–572	71.0%
SIV1	613	555–670	65.1%
SIV2	505	446–563	71.2%
SIV3	459	395–527	73.8%
SNF1	420	375–468	76.1%
SNF2	310	261–360	82.3%
SNF3	193	134–256	89.0%
SAB1	304	264–344	82.7%
SAB2	193	150–233	89.0%
SAB3	80	5–162	95.4%

Supplementary Table 8: Number of bed day reductions. For model abbreviations, see Supplemenatry Figure 1 or the supplementary text.

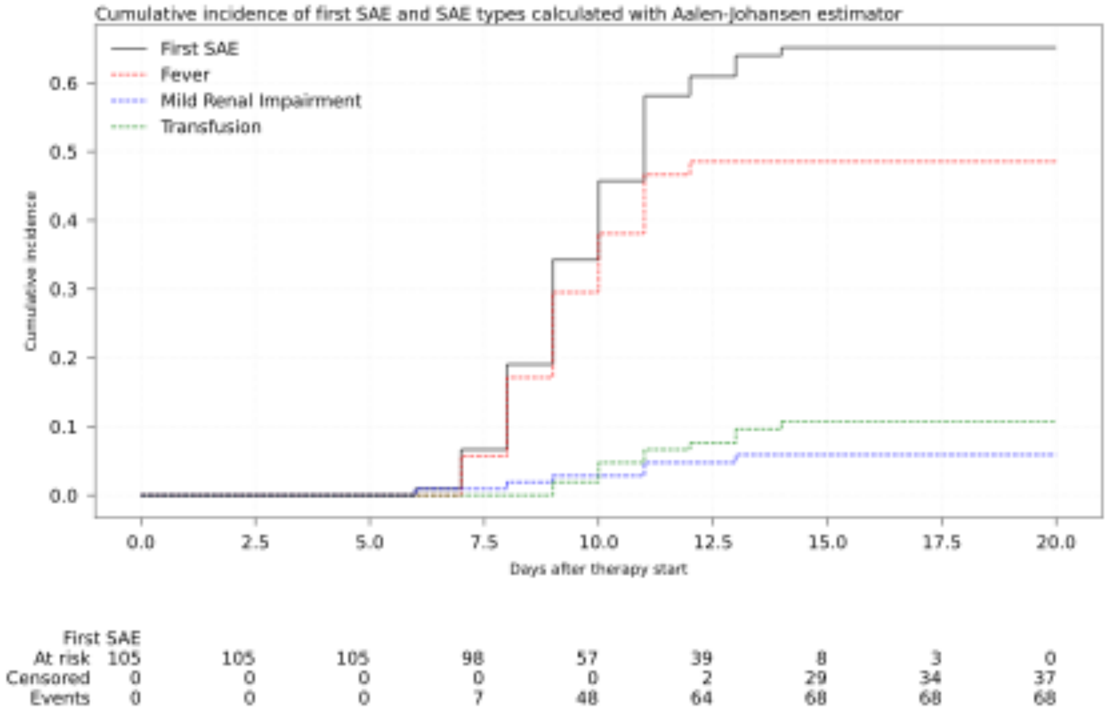

Supplementary Figure 2: An estimation of the cumulative incidence function for individual severe adverse events (SAEs) in a competing risk setting, using the Aalen-Johansen estimator. These calculations are limited to patients who developed SAEs after day 3. Additionally, the combined cumulative incidence function for the first SAE occurrence is provided. Censoring is defined as discharge from the hospital without experiencing any SAE. Mild renal impairment: Serum Creatinine ≥1.2mg/dL irrespective from baseline, triggering supporting IV fluids.

Bibliography

1. Klastersky, J. *et al.* The Multinational Association for Supportive Care in Cancer Risk Index: A Multinational Scoring System for Identifying Low-Risk Febrile Neutropenic Cancer Patients. *J. Clin. Oncol.* **18**, 3038–3051 (2000).
2. Rufibach, K., Beyersmann, J., Friede, T., Schmoor, C. & Stegherr, R. Survival analysis for AdVerse events with VarYing follow-up times (SAVVY): summary of findings and assessment of existing guidelines. *Trials* **25**, 353 (2024).
3. Aalen, O. Nonparametric Estimation of Partial Transition Probabilities in Multiple Decrement Models. *Ann. Stat.* **6**, (1978).

Supplement 5: TRIPOD+AI Checklist Answer Summary: Predicting Adverse Events for Risk-Stratified Chemotherapy-Based Stem Cell Mobilization in Multiple Myeloma

Prepared against the TRIPOD+AI expanded 27-item checklist. Titel and Abstract items are skipped. Items labelled D/E indicate applicability to model development (D) and/or evaluation (E).

Item	Response
Item 3a — Background: context & rationale (D/E)	Inpatient mobilization is common in Germany; shifting appropriate patients to outpatient care could relieve hospital bed constraints. Predictive modelling may enable safe triage and efficient resource planning by forecasting which patients will develop SAEs and when.
Item 3b — Background: target population & intended use (D/E)	Target population: adults with multiple myeloma undergoing chemotherapy-assisted stem cell mobilization and collection. Intended purpose/users: clinicians and bed-management teams to support risk-stratified admission and early scheduling.
Item 3c — Background: health inequalities (D/E)	Outpatient infrastructure may not be universally available and highlights equity and access as ethical considerations for implementation. No sociodemographic characteristics should influence outcome.

Item 4 — Objectives (D/E)	1) Develop and internally validate models to predict occurrence of SAEs (composite and components: neutropenic fever, transfusion, mild renal impairment). 2) Among patients with events, estimate time-to-onset (days) to assist ward scheduling. 3) Simulate alternative admission/treatment scenarios to quantify potential bed-day reductions based on these scenarios. Detailed Scenario descriptions can be found in Supplementary Table 1, and Supplementary Figure 1 and Supplementary Table 8.
Item 5a — Data sources (D/E)	Single-center retrospective cohort derived from clinical records at the University Medical Center Göttingen (UMG). Dataset used for model development and internal evaluation (cross-validation).
Item 5b — Dates (D/E)	Participant accrual: August 2019 – December 2022. Outcomes and laboratory data were collected throughout the inpatient episode until stem-cell collection completion.
Item 6a — Setting (D/E)	Secondary/tertiary care, inpatient hematology/oncology service at UMG, Germany; single center.
Item 6b — Eligibility criteria (D/E)	Adults with confirmed multiple myeloma scheduled for chemotherapy-based stem cell mobilization and subsequent collection; ECOG 0–1; all remission statuses and induction regimens included.

Item 6c — Treatments (D/E)	Chemotherapy mobilization regimens included cyclophosphamide (2.5–4 g/m²) with/without etoposide (100–375 mg/m²) plus G-CSF; treatment indicators and doses were included as predictors where applicable.
Item 7 — Data preparation & quality checks (D/E)	Preprocessing performed within resampling folds to avoid leakage using scikit-learn pipelines. Missing covariates (overall <0.5%) imputed via running averages for repeated measures or population means for singular time points; outcomes did not miss.
Item 8a — Outcome definition & horizon (D/E)	Primary binary outcomes: composite 'any SAE' (CTCAE ≥3 requiring in-hospital care) and specific SAEs—neutropenic fever (>38.2 °C), (erythrocyte) transfusion requirement, mild renal impairment (creatinine ≥1.2 mg/dL triggering IV fluids). Time horizon: inpatient episode during mobilization/collection. For onset forecasting, time (days from therapy start) was modelled among those with events.
Item 8b — Outcome assessors (D/E)	Outcomes derived from clinical documentation and CTCAE grading; assessor are trained doctors.
Item 8c — Blinding of outcome assessment (D/E)	No blinding performed. Design not applicable.
Item 9a — Choice of initial predictors (D)	Three prespecified sets: (1) clinically informed core labs and treatment history; (2) expansion by mutual information; (3) longitudinal extension adding day-3 and day-5 lab values.

Item 9b — Predictor definitions and measurement (D/E)	Baseline labs at admission; therapy details (dose/drugs); longitudinal labs on days 3 and 5 after therapy start. Standardization applied only on training folds where used.
Item 9c — Predictor assessors (D/E)	Laboratory-based predictors or standardized criteria following International Myeloma Working Group (IMWG). Blinding not applicable.
Item 10 — Sample size (D/E)	N=109. No a priori sample-size calculation. All patient of available interval. Event counts: CTCAE ≥3 in 75/109; neutropenic fever 59/109; transfusion 28/109; mild renal impairment 12/109.
Item 11 — Missing data (D/E)	Covariate missingness <0.5%; imputed as above. No missing outcome data. Running average imputation for longitudinal data: imputes with respective patient values. Singular data point imputation of relevant cohort mean: best possible predictor in these cases for cohort.
Item 12a — Data use & partitioning (D)	Development and internal evaluation via k-fold cross-validation (classification: 5-fold; regression: 3-fold). No separate external evaluation set.
Item 12b — Predictor handling (D)	Continuous predictors optionally standardized within folds; no complex transformations performed. Feature sets defined a priori and via mutual information; longitudinal features treated as additional predictors.

Item 12c — Model type, tuning, internal validation (D)	Classifiers: Logistic Regression, Random Forest, Gradient-Boosted Trees/XGBoost, TabPFN. Regressors: Elastic-Net, RF, XGBoost, TabPFN. Hyperparameters tuned in nested CV; Sklearn pipelines used to avoid leakage.
Item 12d — Heterogeneity across clusters (D/E)	Not applicable—single-center dataset; no clustering analyses performed.
Item 12e — Performance measures & plots (D/E)	Classification: accuracy, ROC-AUC, Matthews Correlation Coefficient (MCC). Regression: mean absolute deviation (MAD) and root-mean-squared deviation (RMSD). No calibration performed. Decision curve tuning could be done based on ramification severity estimates of false positive or false negatives.
Item 12f — Model updating (E)	No model updating or recalibration performed.
Item 12g — How predictions were obtained for evaluation (E)	Predictions obtained via scikit-learn/XGBoost/TabPFN model objects within cross-validation pipelines; code is available on request.
Item 13 — Class imbalance (D/E)	Outcomes were moderately to severely imbalanced. Where supported, inverse-frequency class weights were applied during training.
Item 14 — Fairness (D/E)	No explicit fairness audits, bias mitigation, or subgroup performance by sociodemographic variables reported. Equity considerations for outpatient access are discussed qualitatively.

Item 15 — Model output and thresholds (D)	Outputs are predicted probabilities for binary outcomes and continuous day-of-onset for regressors. No prespecified clinical decision thresholds or risk strata were finalized for deployment.
Item 16 — Differences between development and evaluation datasets (D/E)	All data derived from the same center and period; no external evaluation dataset. Therefore, no setting, eligibility, or measurement differences between development and evaluation.
Item 17 — Ethical approval & consent (D/E)	Approved by the UMG Ethics Committee (Göttingen no. 24/1/23). Conducted in accordance with the Declaration of Helsinki and GCP. Consent procedures followed institutional standards.
Item 18a — Funding (D/E)	FS was supported by the Deutsche Forschungsgemeinschaft (DFG, German Research Foundation) - Project-ID 273725443 - SPP 1782, by the DFG - Project-ID 317475864 - SFB 1286, and by the Ministry for Science and Culture of Lower Saxony (MWK) and the Volkswagen Foundation through the program “Niedersächsisches Vorab”. FS acknowledges further support through the Center for Biostructural Imaging of Neurodegeneration, Göttingen, Germany.
Item 18b — Conflicts of interest (D/E)	The authors declare no conflict of interest.
Item 18c — Protocol (D/E)	No registered or public protocol was prepared. Retrospective study.
Item 18d — Registration (D/E)	Not registered. Retrospective study.

Item 18e — Data sharing (D/E)	Patient-level data are not publicly available due to privacy/ethics; may be available from the corresponding author upon reasonable request.
Item 18f — Code sharing (D/E)	Analysis scripts available upon request.
Item 19 — Patient & Public Involvement (PPI) (D/E)	No PPI activities involved.
Item 20a — Participant flow (D/E)	Included N=109 inpatient SCM episodes. Early SAEs (<72h) occurred in 4/109. The remainder either experienced later SAEs before collection or no SAEs; follow-up spanned the inpatient episode until collection. A flow diagram is recommended.
Item 20b — Participant characteristics (D/E)	Report overall and (if applicable) by regimen: age, key baseline labs, prior therapies, mobilization regimen (cyclophosphamide ± etoposide), and counts of outcomes. Missing data were minimal (<0.5%). Differences by key demographics not presented. See Table 1 for a detailed description of the patient cohort.
Item 20c — Development vs evaluation data comparison (E)	Not applicable—no external evaluation set; internal CV used.
Item 21 — Numbers in each analysis (D/E)	Classification used all 109 participants with event counts per outcome (Any SAE: 75; NF: 59; Transfusion: 28; Mild renal impairment: 12). For onset regression, only event-positive patients were included per endpoint; 3-fold CV used due to smaller sample sizes.

Item 22 — Full model specification (D)	Algorithm classes and final hyperparameters are listed, see Supplementary Tables 7 and 8 for more details. Third-party evaluation was not performed.
Item 23a — Performance with uncertainty (D/E)	Reported as mean over folds. Confidence intervals are not reported.
Item 23b — Heterogeneity across clusters (D/E)	Not assessed (single-center dataset).
Item 24 — Model updating (E)	No recalibration or model updating performed.
Item 25 — Interpretation (D/E)	Models suggest some SAEs (e.g., mild renal impairment) are predictable at admission/early labs, while neutropenic fever is harder to predict. Regression prediction error slightly above one day, which is the ideal threshold for an efficient ward bed management. Sensitivity analysis suggests model improvements when sample size can be increased.
Item 26 — Limitations (D/E)	Single-center study; modest sample size; internal validation only; class imbalance; regression restricted to cases with events.
Item 27a — Usability — handling poor quality/unavailable inputs (D)	All data was checked for plausibility by specialized doctors. Imputations are plausible due to used procedure.
Item 27b — Usability — user interaction & expertise (D)	Intended users are clinicians (specialization in hematology) and or bed-managers of hematological wards; A risk-threshold policy and workflow integration can be defined prior to deployment.

Item 27c — Next steps & generalizability (D/E)	External, multicenter validation; addition of calibration and clinical-utility (decision-curve) analyses; prospective impact evaluation; definition of actionable risk thresholds and guardrails for outpatient triage.

Supplementary Table 9: Answers to the TRIPOD+AI checklist for the used models in "Predicting Adverse Events for Risk-Stratified Chemotherapy-Based Stem Cell Mobilization in Multiple Myeloma".

Source TRIPOD+AI:

Collins G S, Moons K G M, Dhiman P, Riley R D, Beam A L, Van Calster B et al. TRIPOD+AI statement: updated guidance for reporting clinical prediction models that use regression or machine learning methods BMJ 2024; 385 :e078378 doi:10.1136/bmj-2023-078378
